# High expression of B4GALT1 is associated with poor prognosis in acute myeloid leukemia

**DOI:** 10.3389/fgene.2022.882004

**Published:** 2022-12-09

**Authors:** Zhihong Ren, Xiaoyu Huang, Qing Lv, Yiming Lei, Haiqiang Shi, Fanping Wang, Mingyong Wang

**Affiliations:** ^1^ School of Laboratory Medicine, Xinxiang Medical University, Xinxiang, China; ^2^ Henan Key Laboratory of Immunology and Targeted Drug, School of Laboratory Medicine, Xinxiang Medical University, Xinxiang, China; ^3^ School of Nursing and Health, Hennan University, Kaifeng, China; ^4^ School of Public Health, Xinxiang Medical University, Xinxiang, China; ^5^ Xinxiang Key Laboratory of Immunoregulation and Molecular Diagnostics, Xinxiang Medical University, Xinxiang, China

**Keywords:** B4GALT1, acute myeloid leukemia, biomarker, immune infiltration, prognosis

## Abstract

Acute myeloid leukemia is the most prevalent type of leukemia in adults and is prone to relapse and chemoresistance, with a low long-term survival rate. Therefore, the identification of quality biomarkers constitutes an urgent unmet need. High expression of beta-1,4-galactosyltransferase 1 (B4GALT1) has been observed in several cancer types; however, its function in acute myeloid leukemia has rarely been studied. Therefore, our study obtained gene expression data from The Cancer Genome Atlas (TCGA) database to analyze the relationship between B4GALT1 and LAML. We compared the expression of B4GALT1 in LAML and healthy samples using the Wilcoxon rank-sum test. Furthermore, the association between B4GALT1 and survival rates was investigated using Kaplan-Meier analysis and Cox regression. The nomogram obtained by Cox analysis predicts the effect of B4GALT1 on the prognosis. To assess B4GALT1-related genes’ enrichment pathway and function and the correlation between B4GALT1 and immune features, GO/KEGG, protein-protein interaction network, and single sample gene set enrichment analysis were used. In addition, B4GALT1-specific siRNAs were used to verify the effect of B4GALT1 on apoptosis. The results showed that B4GALT1 is overexpressed in LAML and has some reference value in the diagnostic and prognostic assessment of LAML. Moreover, functional enrichment showed that B4GALT1 and its 63 associated genes were closely associated with the negative regulation of the apoptotic signaling pathway. Silencing B4GALT1 significantly promoted apoptosis. In addition, B4GALT1 expression was positively correlated with the infiltration levels of macrophages, regulatory T-cell (Tregs), and Th17 cells; in contrast, B4GALT1 expression was negatively correlated with the infiltration levels of T helper cells, Mast cells, and NK cells. In conclusion, our study shows that B4GALT1 may play a vital role in the occurrence of LAML.

## Introduction

Acute myeloid leukemia (LAML) is a malignant clonal disease of hematopoietic stem cells, characterized by the proliferation of leukemic cells in the bone marrow and other hematopoietic tissues due to uncontrolled cell cycle, blocked apoptosis, impaired differentiation, and other mechanisms, which in turn infiltrate other tissues and organs (Dohner et al., 2015; Briot et al., 2018; Rudat et al., 2018). Many efforts have been aimed at improving the prognosis of LAML, but since 1970, the standard treatment for most types of LAML has not changed. The primary treatment drug, cytarabine, usually develops drug resistance; one of the reasons is that tumor immune cells evade the drug-induced apoptosis or autophagy. Therefore, most patients will eventually relapse. Recently, the development of molecularly targeted drugs has prolonged the survival of LAML patients and improved clinical outcomes, highlighting the need for new targeted therapies (Estey, 2016). Therefore, exploring more effective biomarkers may provide a new molecular therapeutic strategy for LAML.

Beta-1,4-galactosyltransferase 1 (B4GALT1) is a member of the β-1, 4-galactosyltransferase gene family that encodes a type II membrane-bound glycoprotein that transfers galactose to a similar receptor sugar in the β-1, 4-bonded form (Youakim et al., 1994). Because the promoter region upstream of the B4GALT1 start site contains the ubiquitous transcription factor Sp1, it has long been thought to be a housekeeping gene (Rajput et al., 1996). B4GALT1 plays a vital role in many biological processes, including cell growth, sperm-egg interaction, cell adhesion, migration, and brain development (Nakamura et al., 2001; Nixon et al., 2001; Qasba et al., 2008; Han et al., 2010). Recently, there has been increasing evidence that abnormal expression of B4GALT1 can lead to the development and malignant transformation of various tumors. Tang et al. (2013) found that β1,4-galactosyltransferase one affects the growth and apoptosis of hepatocellular carcinoma cells by regulating EGFR. Estrogen and arachidonic acid have been reported to promote proliferation and cell adhesion of human breast cancer cells by inducing B4GALT1 expression (Choi et al., 2012; Villegas-Comonfort et al., 2012). In glioblastoma, B4GALT1 regulates apoptosis and autophagy, enhancing tumor proliferation, migration, and invasion (Wang et al., 2020). However, the role of B4GALT1 in LAML and its prognostic value have rarely been reported.

Our study aims to use gene expression data obtained from the TCGA database to clarify the potential biological effects between B4GALT1 and LAML patients. Secondly, Kaplan-Meier and prognostic nomogram models were used to explore B4GALT1’s potential diagnostic and prognostic value. Moreover, systematically assess the importance of B4GALT1 in LAML through bioinformatics and statistical methods. Our study shows that B4GALT1 plays a critical role in the carcinogenesis and progression of LAML, and it may be used as a biomarker to predict patient survival.

## Materials and methods

### Data source

Messenger RNA (mRNA) expression data were obtained from the TCGA website (https://cancergenome.nih.gov), which contains gene expression data (HTSeq-FPKM) and clinical information for 151 LAML patients. Next, the Level-3 HTSeq-FPKM data was converted to transcripts per million reads (TPM). Ethical approval and patient informed permission are unnecessary because our study follows the TCGA and GTEx guidelines.

### Timer database analysis

TIMER (http://timer.cistrome.org/) (Li et al., 2017) is an online analysis tool with multiple functions. It can explore gene expression levels in multiple tumor para-cancer and tumor tissues in the TCGA dataset.

### Prognostic model generation and prediction

A nomogram was built based on independent prognostic indicators derived in multivariate analysis to customize the estimated survival probabilities of 1, 3, and 5 years. The RMS package (version 6.2–0) was employed to generate nomograms that included significant clinical characteristics and calibration plots. The calibration curves were visually assessed by comparing the nomogram-predicted probabilities to the observed rates, with the 45° line representing the best predictive values. The discrimination of the nomogram was determined using the concordance index (C-index), which was generated using a bootstrap technique with 1000 resamples. Moreover, the predictive accuracies of the nomogram and various clinicopathological prognostic factors were compared using the C-index and ROC analyses.

### GO and KEGG pathway enrichment analysis

To understand the biological processes and pathways in which B4GALT1-related genes may be involved, we performed Gene Ontology (GO) and KEGG pathway analyses on the top 600 genes associated with B4GALT1 and 63 genes associated with LAML prognosis in LAML using the ClusterProfiler (version 3.14.3) package in R (Yu et al., 2012). Biological process (BP), cellular component (CC), and molecular function (MF) categories were included in the GO analysis.

### Protein–protein interaction network

The Search Tool for the Retrieval of Interacting Genes (STRING), a database of known and predicted protein-protein interactions (PPI), was used to construct a PPI network for the differentially expressed genes (DEGs) (Szklarczyk et al., 2019). A composite score threshold of 0.4 was set as the cutoff criterion. The network is imported into the Cytoscape 3.7.0 application for visualization.

### Immune cell infiltration estimation with ssGSEA

Immune infiltration analysis of B4GALT1 was conducted by ssGSEA using the GSVA package in R (3.6.3) (Hanzelmann et al., 2013). Marker genes for the 24 immune cell types used in ssGSEA were obtained from Bindea and Gabriela et al. (Bindea et al., 2013). The association between B4GALT1 and the enrichment fraction of each immune cell was investigated using the Spearman correction. The enrichment scores of high- and low-B4GALT1 expression groups were analyzed with the Wilcoxon rank-sum test.

### Cell culture and transfection

The Department of Hematology donated the human leukemia cell line KG1a, Zhujiang Hospital, Southern Medical University, and K562 cells were purchased from the Institute of Hematology & Blood Diseases Hospital Chinese Academy of Medical Sciences (Tianjin, China). All cell lines were cultured in RPMI 1640 (Gibco, United States) medium containing 10% fetal bovine serum (Gibco) in a 37°C, 5% CO2 incubator. We established two ADR sublines, KG1A/ADR and K562/ADR, from the corresponding parental cell lines by stepwise exposing cells to doxorubicin at concentrations increasing from 0.02 ug/ml to one ug/ml for 3 months. B4GALT1 small interfering RNA as well as the corresponding control RNA were transfected into logarithmic growth phase cells. Transfection was performed using Lipofectamine 3000 transfection reagent (Invitrogen, United States) according to the manufacturer’s protocol. B4GALT1 small interfering RNA as well as the corresponding control RNA were designed by RiboBio.

### Quantitative reverse transcription PCR (RT-qPCR)

The total RNA was extracted from cells with Trizol reagents. According to the manufacturer’s instructions, cDNA was synthesized from 1 μg of mRNA with a high-capacity cDNA reverse transcription kit. Subsequently, cDNA was amplified by QPCR with the SYBR Premix Ex Taq kit according to the manufacturer’s instructions using the ABI7300 Sequence Detection System. PCR conditions were as follows: one cycle at 95°C for 3 min, followed by 40 cycles at 95°C for 30 s and 64°C for 1 min. All assays were performed in triplicate and were calculated based on the 2^−ΔΔCT^ method. For qRT-PCR, the following primers were used: human B4GALT1, 5′- CCA​GGC​GGG​AGA​CAC​TAT​ATT-3′ (Forward) and 5′-CAC​CTG​TAC​GCA​TTA​TGG​TCA​T-3′ (Reverse); human β-actin, 5′-AGA​GCT​ACG​AGC​TGC​CTG​AC-3′ (Forward) and 5′- AGC​ACT​GTG​TTG​GCG​TAC​AG-3′ (Reverse).

### CCK-8

To assess proliferation, K562 and HL60 cells transfected with B4GALT1 siRNA were seeded in a 96-well plate and cultured in a 37°C incubator. At the same time point for the next 4 days, add 10 μl of Cell Counting Kit-8 to each well and incubate for 3 h. Absorbance (450 nm) was measured by a Microplate Reader (Thermo Scientific).

### Western blotting

Total protein was extracted from the cells using ice-cold RIPA lysis buffer and the protein concentration was measured using the BCA Protein Assay Kit. 30 µg total proteins were applied on 10% SDS-PAGE and transferred onto 0.45 mm PVDF membranes (Millipore, United States). The membranes were blocked with 5% non-fat milk for 2 h at room temperature, and then incubated with primary antibodies overnight at 4°C. After washing with TBST buffer three times, the membranes were incubated with secondary HRP-conjugated antibodies for 1 h at room temperature. Capture specific bands using the ECL detection system. The primary antibodies were anti-B4GALT1 (Abcam, ab121326, 1:1000), anti-Bcl-2(Proteintech, 12789-1-AP, 1:1000), anti-Bax (Proteintech, 50,599-2-lg, 1:1000), anti-C-caspase (Proteintech, 19677-1-AP, 1:1000) and anti-β-actin (Proteintech, 20536-1-AP, 1:1000).

### Cell apoptosis assay

Cell apoptosis was performed with Annexin V-fluorescein isothiocyanate (FITC)/propidium iodide (PI) staining kit (Beyotime Biotechnology, Jiangsu, China). According to the manufacturer’s instruction, the cells were seeded in 6-well plates and incubated for 24 h, followed by transfection for 48 h. Cells were collected, centrifuged, washed twice with PBS, resuspended in 400 µl binding buffer and then incubated with 5 µl Annexin V-FITC and 10 µl PI at room temper-ature for 15 min in the dark. Cells were analyzed for fluorescence with flow cytometer (BD Corp).

### Statistical analysis

R (Version 3.6.3) was used to perform all statistical analyses and visualizations. The Wilcoxon rank sum test was used for the B4GALT1 expression in unpaired samples. The connection between clinical-pathologic characteristics and B4GALT1 expression was evaluated using the Kruskal-Wallis test and the Wilcoxon signed-rank test. Cox regression analysis and the Kaplan-Meier method evaluated the prognostic factors. The influence of B4GALT1 expression on survival and other clinical information was compared using multivariate Cox analysis. In addition, ROC analysis was performed on the pROC software package (Version 1.17.0.1) to evaluate the effectiveness of B4GALT1 expression in distinguishing LAML from healthy samples. The calculated AUC value ranges from 0.5 to one and implies a discriminative potential of 50–100%. *p* values <0.05 were considered statistically significant in all tests.

## Results

### The mRNA expression level of B4GALT1 in pan-cancers and LAML

**FIGURE 1:**
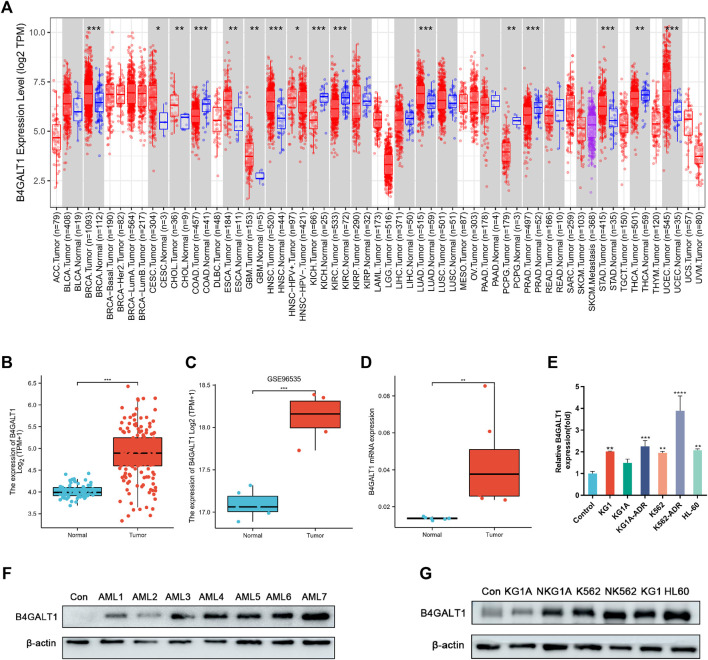
The expression levels of B4GALT1 in different human cancers. **(A)** TIMER was used to detect the expression levels of B4GALT1 in different tumors in The Cancer Genome Atlas (TCGA) database. **(B)** Expression level of B4GALT1 in paired normal and LAML samples. **(C)** Expression of B4GALT1 in tumor and normal samples from the GSE96535 dataset in the Gene Expression Omnibus (GEO) database. **(D)** Quantitative mRNA expression of B4GALT1 by qRT-PCR in LAML patient samples (*n* = 8) and controls (*n* = 6). **(E)** Transcriptional level of B4GALT1 in different LAML cell lines. **(F–G)** Western blot detecting the protein expression level of B4GALT1 in patients and different LAML cell lines. Analysis between two groups: Wilcoxon Rank sum test; NS:*p* ≥ 0.05; *, *p* < 0.05; **, *p* < 0.01; ***, *p* < 0.001.

To explore the expression levels of B4GALT1 in normal and tumor tissues, we analyzed the expression levels of B4GALT1 mRNA in different tumor and normal tissues using the TIMER database. We found that B4GALT1 is highly expressed in eight tumor types and low in six tumor types (Figure 1A). Furthermore, due to the lack of normal control samples of LAML in the TCGA database, we further integrated the healthy tissue data from the GTEx database and performed Wilcoxon rank sum test for the expression of B4GALT1 in LAML. The results showed that B4GALT1 was overexpressed in LAML (Figure 1B).

To further verify the relationship between B4GALT1 and LAML, we validated B4GALT1 expression in LAML and the prognosis by GSE96535 and GSE71014 in the GEO (Gene Expression Omnibus) database. The results were as predicted in the TCGA database, B4GALT1 was highly expressed in LAML, and high B4GALT1 expression was associated with poor prognosis in LAML patients (Figure 1C, Supplementary Figure S1). At the same time, we collected bone marrow specimens from six healthy individuals and eight LAML patients and similarly validated that B4GALT1 was highly expressed in LAML (Figure 1D, Figure 1F). In addition, the expression levels of B4GALT1 in LAML cell lines were detected by QPCR and Western blotting. The results showed that the expression levels of B4GALT1 were significantly higher in LAML cells and drug-resistant cells than in the control (Figure 1E, Figure 1G).

### Association of B4GALT1 expression with clinicopathological status

The clinicopathological characteristics of all LAML patients are demonstrated, as shown in Table 1. The study included 68 women and 83 men. According to the median value of B4GALT1 expression (log2 (TPM+1)), B4GALT1 was low in 75 LAML patients and high in the remaining 76 patients. Correlation analysis suggested that B4GALT1 expression was significantly correlated with Cytogenetic risk (*p* = 0.003), FAB classification (*p* < 0.001), NPM1 mutation (*p* = 0.023), and Age (*p* = 0.006). In addition, the incidence of t (8; 21) and t (15; 17) was lower in the B4GALT1 high expression group (*p* < 0.007).

**TABLE 1:** Association between B4GALT1 expression and clinicopathologic features in LAML samples from the TCGA database.

Characteristic	Levels	Low expression of B4GALT1	High expression of B4GALT1	*p*
N		75	76	
Gender, n (%)	Female	38 (25.2%)	30 (19.9%)	0.223
	Male	37 (24.5%)	46 (30.5%)	
Race, n (%)	Asian	0 (0%)	1 (0.7%)	1.000
	Black or African American	6 (4%)	7 (4.7%)	
	White	67 (45%)	68 (45.6%)	
Age, n (%)	<=60	49 (32.5%)	39 (25.8%)	0.114
	>60	26 (17.2%)	37 (24.5%)	
WBC count (x10^9/L), n (%)	<=20	35 (23.3%)	42 (28%)	0.417
	>20	39 (26%)	34 (22.7%)	
BM blasts (%), n (%)	<=20	24 (15.9%)	36 (23.8%)	0.078
	>20	51 (33.8%)	40 (26.5%)	
PB blasts (%), n (%)	<=70	37 (24.5%)	35 (23.2%)	0.810
	>70	38 (25.2%)	41 (27.2%)	
Cytogenetic risk, n (%)	Favorable	23 (15.4%)	8 (5.4%)	0.003
	Intermediate	38 (25.5%)	44 (29.5%)	
	Poor	12 (8.1%)	24 (16.1%)	
FAB classifications, n (%)	M0	9 (6%)	6 (4%)	<0.001
	M1	15 (10%)	20 (13.3%)	
	M2	27 (18%)	11 (7.3%)	
	M3	14 (9.3%)	1 (0.7%)	
	M4	6 (4%)	23 (15.3%)	
	M5	2 (1.3%)	13 (8.7%)	
	M6	0 (0%)	2 (1.3%)	
	M7	1 (0.7%)	0 (0%)	
Cytogenetics, n (%)	Normal	30 (22.2%)	39 (28.9%)	0.007
	+8	3 (2.2%)	5 (3.7%)	
	del (5)	1 (0.7%)	0 (0%)	
	del (7)	1 (0.7%)	5 (3.7%)	
	inv (16)	3 (2.2%)	5 (3.7%)	
	t (15; 17)	10 (7.4%)	1 (0.7%)	
	t (8; 21)	7 (5.2%)	0 (0%)	
	t (9; 11)	0 (0%)	1 (0.7%)	
	Complex	12 (8.9%)	12 (8.9%)	
FLT3 mutation, n (%)	Negative	47 (32%)	55 (37.4%)	0.259
	Positive	26 (17.7%)	19 (12.9%)	
IDH1 R132 mutation, n (%)	Negative	70 (47%)	66 (44.3%)	0.256
	Positive	4 (2.7%)	9 (6%)	
IDH1 R140 mutation, n (%)	Negative	70 (47%)	67 (45%)	0.152
	Positive	3 (2%)	9 (6%)	
IDH1 R172 mutation, n (%)	Negative	73 (49%)	74 (49.7%)	0.497
	Positive	0 (0%)	2 (1.3%)	
RAS mutation, n (%)	Negative	71 (47.3%)	71 (47.3%)	0.719
	Positive	3 (2%)	5 (3.3%)	
NPM1 mutation, n (%)	Negative	64 (42.7%)	53 (35.3%)	0.023
	Positive	10 (6.7%)	23 (15.3%)	
Age, meidan (IQR)		51 (36.5, 63)	60 (48, 68)	0.006

**FIGURE 2:**
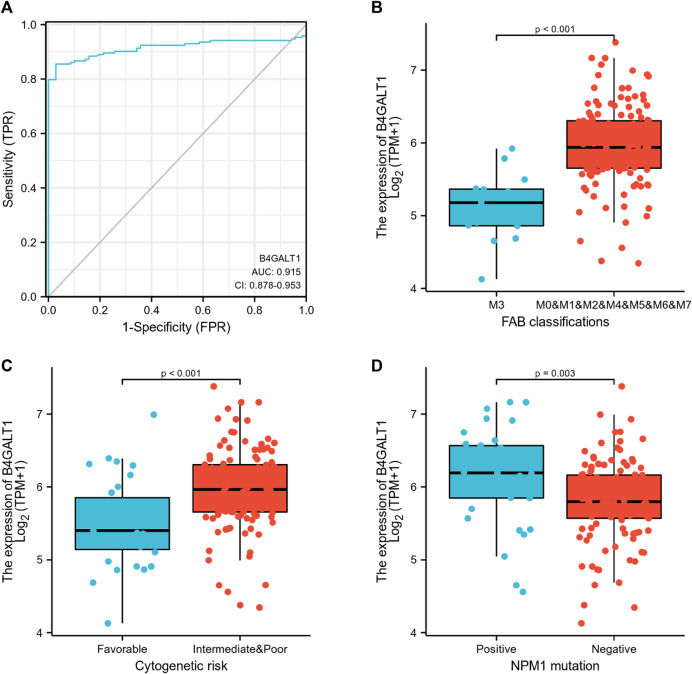
Association between B4GALT1 expression and clinical features. **(A)** ROC analysis of B4GALT1 shows promising discrimination power between tumor and normal tissues. **(B)** FAB classification; **(C)** cytogenetics risk; **(D)** NPM1 mutation.

Logistic regression analysis was used to validate further the relationship between B4GALT1 expression as a categorical dependent variable and clinicopathological features of LAML. The results showed that overexpression of B4GALT1 was significantly and positively associated with unfavorable cytogenetic risk (dominance ratio [OR], 0.256; *p* = 0.002) and NPM1 mutation (OR, 2.777; *p* = 0.015) (Table 2). Besides, ROC curve analysis was used to assess the potential value of B4GALT1 in distinguishing LAML patients from healthy individuals, with an AUC of 0.915 indicating that B4GALT1 showed biomarker potential (Figure 2A). Furthermore, Wilcoxon Rank SUM test results showed that high expression of B4GALT1 was significantly related to FAB classification (non-M3 type; *p* < 0.001), cytogenetic risk (intermediate/poor; *p* < 0.001), and mutated nucleophosmin one gene (NPM1) mutation (positive; *p* = 0.003) (Figures 2B–D).

**TABLE 2:** The relationship between the clinicopathological factors of LAML and B4GALT1 expression was determined by the logistic analysis.

Characteristics	Total(N)	Odds ratio (OR)	*p*-Value
WBC count (x10^9/L) (>20 vs. <=20)	150	0.726 (0.381–1.379)	0.330
PB blasts (%) (>70 vs. <=70)	151	1.141 (0.602–2.166)	0.687
BM blasts (%) (>20 vs. <=20)	151	0.523 (0.267–1.009)	0.055
Cytogenetic risk (Favorable vs. Poor& Intermediate)	149	0.256 (0.100–0.597)	0.002
FLT3 mutation (Positive vs. Negative)	147	0.624 (0.305–1.263)	0.193
IDH1 R132 mutation (Positive vs. Negative)	149	2.386 (0.739–9.149)	0.164
IDH1 R140 mutation (Positive vs. Negative)	149	3.134 (0.892–14.580)	0.097
RAS mutation (Positive vs. Negative)	150	1.667 (0.394–8.372)	0.495
NPM1 mutation (Positive vs. Negative)	150	2.777 (1.243–6.590)	0.015
			

### High expression of B4GALT1 is an independent prognostic factor in LAML patients

**FIGURE 3:**
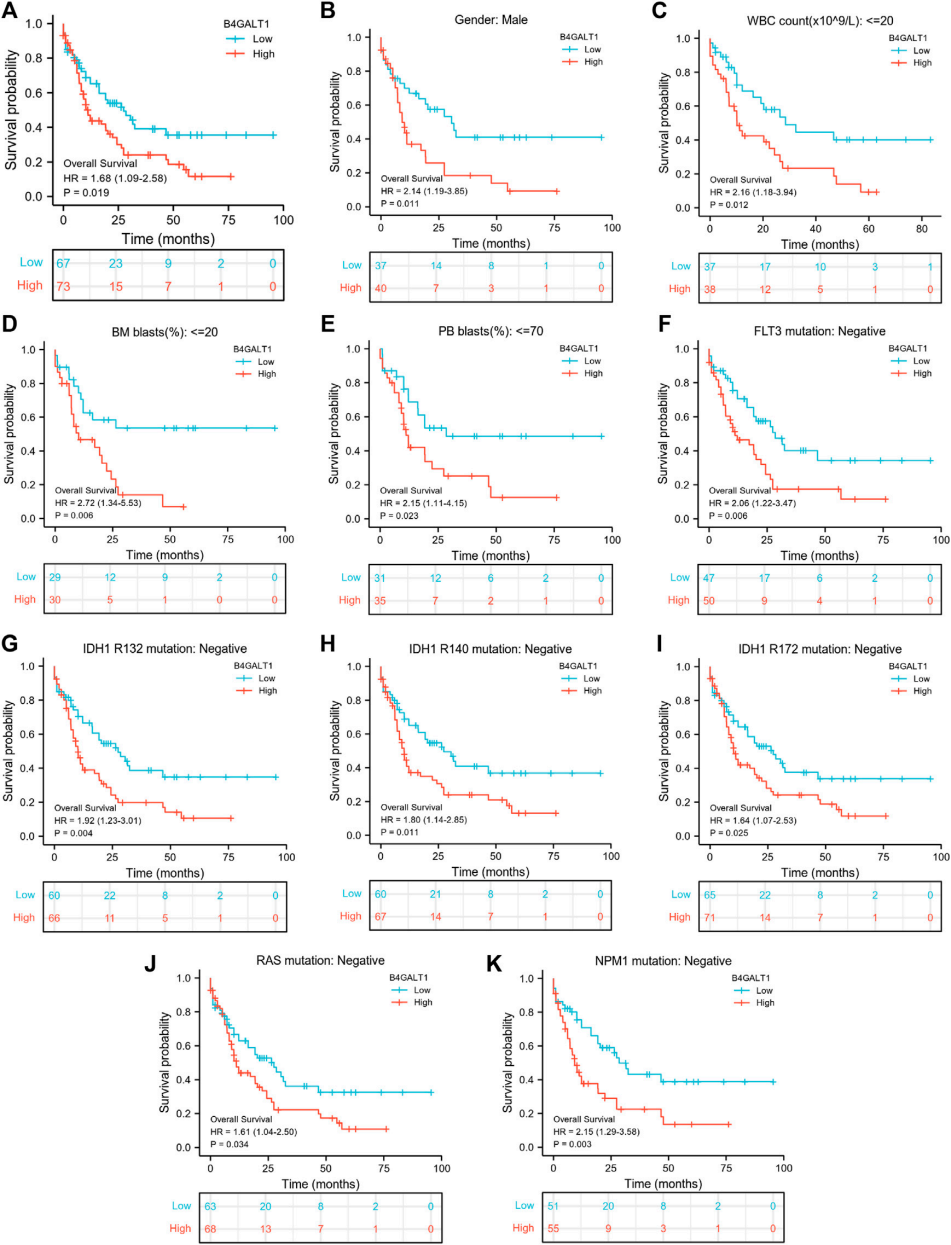
Kaplan-Meier survival curves compare B4GALT1’s high and low expression in LAML patients. **(A)** Overall survival. **(B)** Kaplan-Meier curves in LAML patients with Male. **(C)** Kaplan-Meier curves in LAML patients with WBC counts <= 20 × 10^9^/L. **(D)** Kaplan-Meier curves in LAML patients with BM blasts <20%. **(E)** Kaplan-Meier curves in LAML patients with PB blasts ≤70%. **(F–K)** Kaplan-Meier curves in subgroups with FLT3 mutation-negative, IDH1 R132 mutation-negative, IDH1 R140 mutation-negative, R172 mutation-negative, RAS mutation-negative, and NPM1 mutation-negative in LAML patients.

A Kaplan-Meier analysis was performed to determine whether B4GALT1 expression affects patient survival. The results show that, compared with the low B4GALT1 expression group, high B4GALT1 expression was more strongly related to the poor prognosis of LAML (HR = 1.68, *p* = 0.019) (Figure 3A). Subgroup analysis showed that a high B4GALT1 expression was significantly correlated with poor prognosis in LAML in the following cases: male (HR = 2.14, *p* = 0.011), WBC count (≤20 × 10^9^/L) (HR = 2.16, *p* = 0.012), BM blasts ≤20% (HR = 2.72, *p* = 0.006), PB blasts ≤70% (HR = 2.15, *p* = 0.023), FLT3 mutation-negative (HR = 2.06, *p* = 0.006), IDH1 R132 mutation-negative (HR = 1.92, *p* = 0.004), R140 mutation-negative (HR = 1.80, *p* = 0.011), R172 mutation-negative (HR = 1.64, *p* = 0.025), RAS mutation-negative (HR = 1.61, *p* = 0.034), and NPM1 mutation-negative (HR = 2.15, *p* = 0.003) (Figures 3B–K).

In addition, we used univariate Cox analysis to evaluate the link between B4GALT1 expression and clinical features of LAML patients to understand better the importance and mechanisms of B4GALT1 expression in LAML. The results show that B4GALT1 (high-vs. low-, *p* = 0.019), cytogenetic risk (favorable vs. poor and intermediate, *p* < 0.001) and age (>60 vs. ≤ 60, *p* < 0.001) (Table 3) are Predictors of poor overall survival (OS).

**TABLE 3:** Univariate and multivariate Cox’s regression analysis of factors associated with OS in LAML.

Characteristics	Total(N)	Univariate analysis		Multivariate analysis	
		Hazard ratio (95% CI)	*p*-Value	Hazard ratio (95% CI)	*p*-Value
WBC count (x10^9/L)	139				
<=20	75	References			
>20	64	1.161 (0.760–1.772)	0.490		
BM blasts (%)	140				
<=20	59	References			
>20	81	1.165 (0.758–1.790)	0.486		
PB blasts (%)	140				
<=70	66	References			
>70	74	1.230 (0.806–1.878)	0.338		
Cytogenetic risk	138				
Poor&Intermediate	107	References			
Favorable	31	0.312 (0.160–0.606)	<0.001	0.434 (0.216–0.873)	0.019
Age	140				
<=60	79	References			
>60	61	3.333 (2.164–5.134)	<0.001	2.826 (1.800–4.439)	<0.001
Race	138				
Asian&Black or African American	11	References			
White	127	1.200 (0.485–2.966)	0.693		
Gender	140				
Female	63	References			
Male	77	1.030 (0.674–1.572)	0.892		
FLT3 mutation	136				
Negative	97	References			
Positive	39	1.271 (0.801–2.016)	0.309		
IDH1 R132 mutation	138				
Negative	126	References			
Positive	12	0.588 (0.238–1.452)	0.249		
IDH1 R140 mutation	138				
Negative	127	References			
Positive	11	1.131 (0.565–2.264)	0.727		
IDH1 R172 mutation	138				
Negative	136	References			
Positive	2	0.610 (0.085–4.385)	0.623		
RAS mutation	139				
Negative	131	References			
Positive	8	0.643 (0.235–1.760)	0.390		
NPM1 mutation	139				
Negative	106	References			
Positive	33	1.137 (0.706–1.832)	0.596		
B4GALT1	140				
Low	67	References			
High	73	1.677 (1.090–2.579)	0.019	1.267 (0.802–2.002)	0.311

### Construction and validation of a nomogram based on the B4GALT1

**FIGURE 4:**
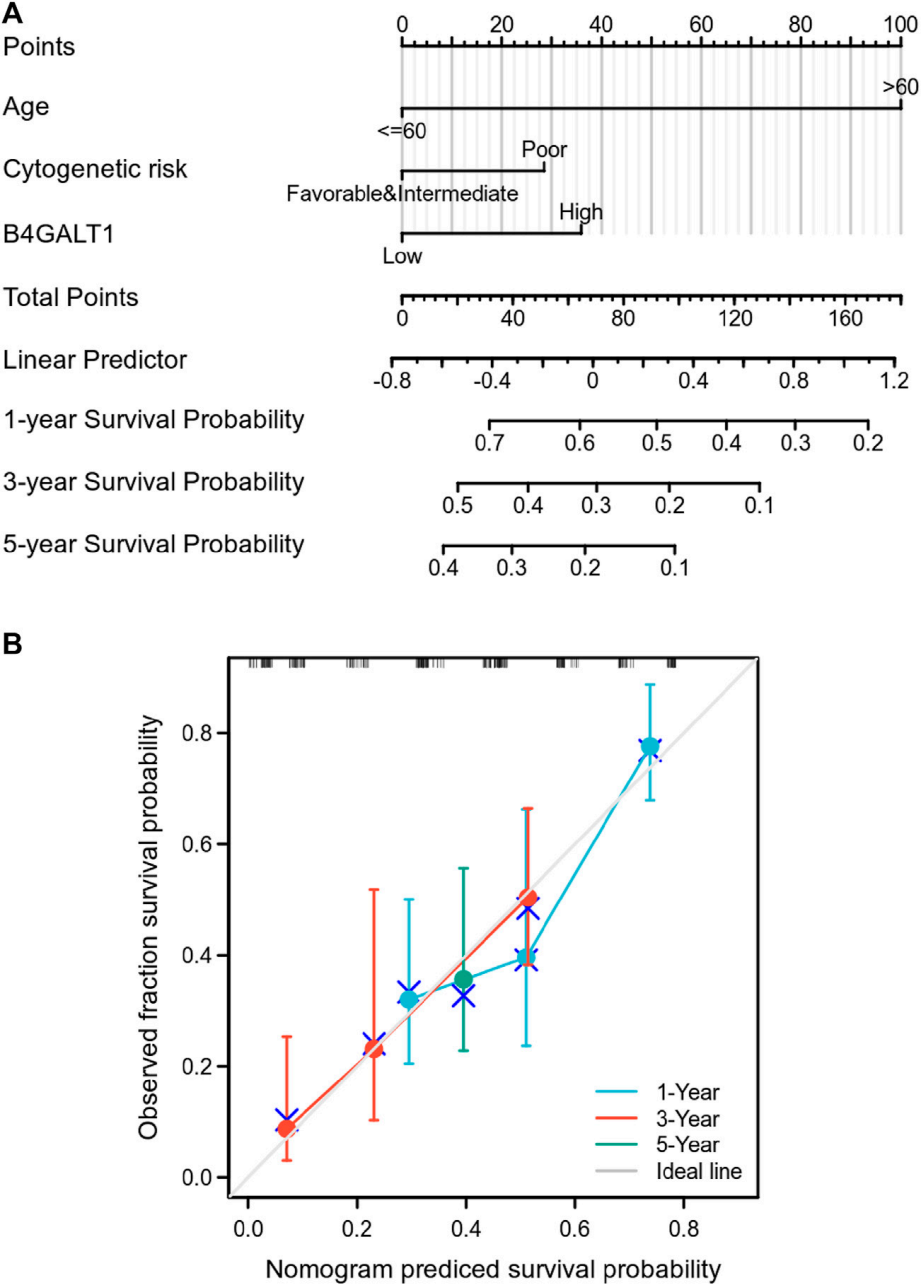
Relationship between B4GALT1 and other clinical factors with overall survival (OS). **(A)** Nomogram for predicting the probability of 1, 3, and 5-year OS for LAML patients. **(B)** Calibration plot of the nomogram for predicting the OS likelihood.

To predict the prognosis of LAML patients more accurately, the RMS R package is used to construct a nomogram based on the results of Cox regression analysis to predict the probability of patient survival at 1, 3, and 5 years (Figure 4A). All patients’ observation findings were compatible with the nomogram calibration curve’s prediction results (Figure 4B).

### Differential genes correlated with B4GALT1 in LAML

**FIGURE 5:**
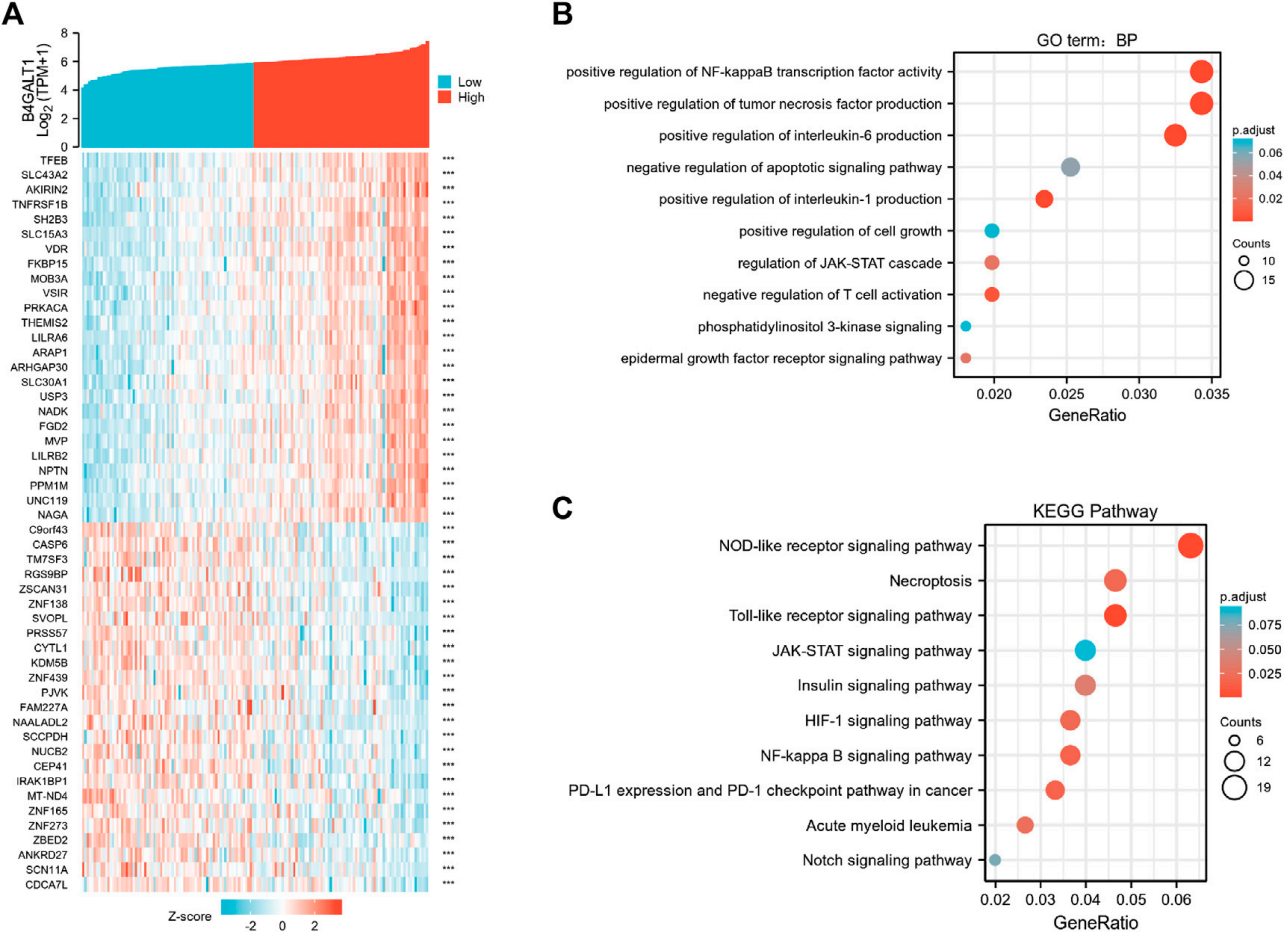
Functional clustering analysis of B4GALT1-related genes. **(A)** Heatmap showing the top 50 genes in acute myeloid leukemia (LAML) that were positively and negatively related to B4GALT1. Red represents positively related genes, and blue represents negatively related genes. **(B)** Enriched GO terms in the “biological process” category; **(C)** KEGG pathway annotations. The B4GALT1-axis represented the proportion of DEGs, and the *Y*-axis represented different categories. The different colors indicate different *p* values, and the different sizes represent gene numbers.

To elucidate the role and significance of B4GALT1 expression in LAML, we examined the differentially expressed genes and gene co-expression pattern of B4GALT1 in LAML. The volcano plot showed 756 up-regulated genes and 553 down-regulated genes, which were statistically significant between the two groups (|log fold change (logFC)| > 1.5, *p*.adj <0.05) (Supplementary Figure S2). Heatmap shows the top 25 genes positively correlated with B4GALT1 and the bottom 25 negatively correlated genes (Figure 5A). We used the ClusterProfiler package to identify B4GALT1-related genes’ biological processes and pathways (top 600). BP analysis showed that these related genes were mainly enriched in negative regulation of apoptotic signaling pathway, positive regulation of interleukin-6 production, and positive regulation of cell growth (Figure 5B). The results of KEGG analysis revealed that these related genes were mainly involved in the NOD-like receptor signaling pathway, Necroptosis, and Toll-like receptor signaling pathway (Figure 5C).

**FIGURE 6:**
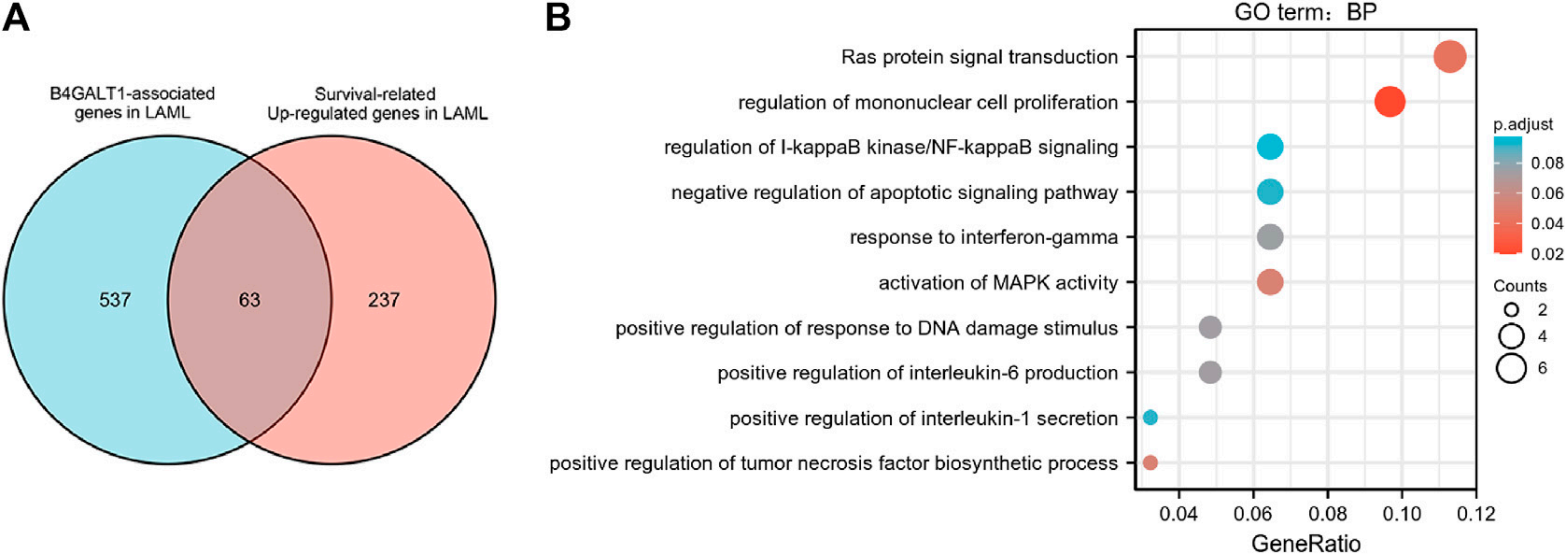
Functional clustering analysis of B4GALT1-related genes. **(A)** Venn diagram of B4GALT1-related genes and survival-related upregulated genes in LAML. **(B)** GO enrichment analysis of B4GALT1-related genes and LAML survival-related genes in LAML.

To identify genes with the same regulatory direction in high B4GALT1 and non-surviving patients, we intersected the 600 highest B4GALT1-related genes with 300 survival-related genes in LAML, detecting a total of 63 genes associated with B4GALT1 and LAML survival-related genes (Figure 6A). Further GO functional enrichment of these 63 genes showed that DEGs were significantly enriched in negative regulation of apoptotic signaling pathway, activation of MAPK activity, and regulation of I-kappaB kinase/NF-kappaB signaling (Figure 6B).

**FIGURE 7:**
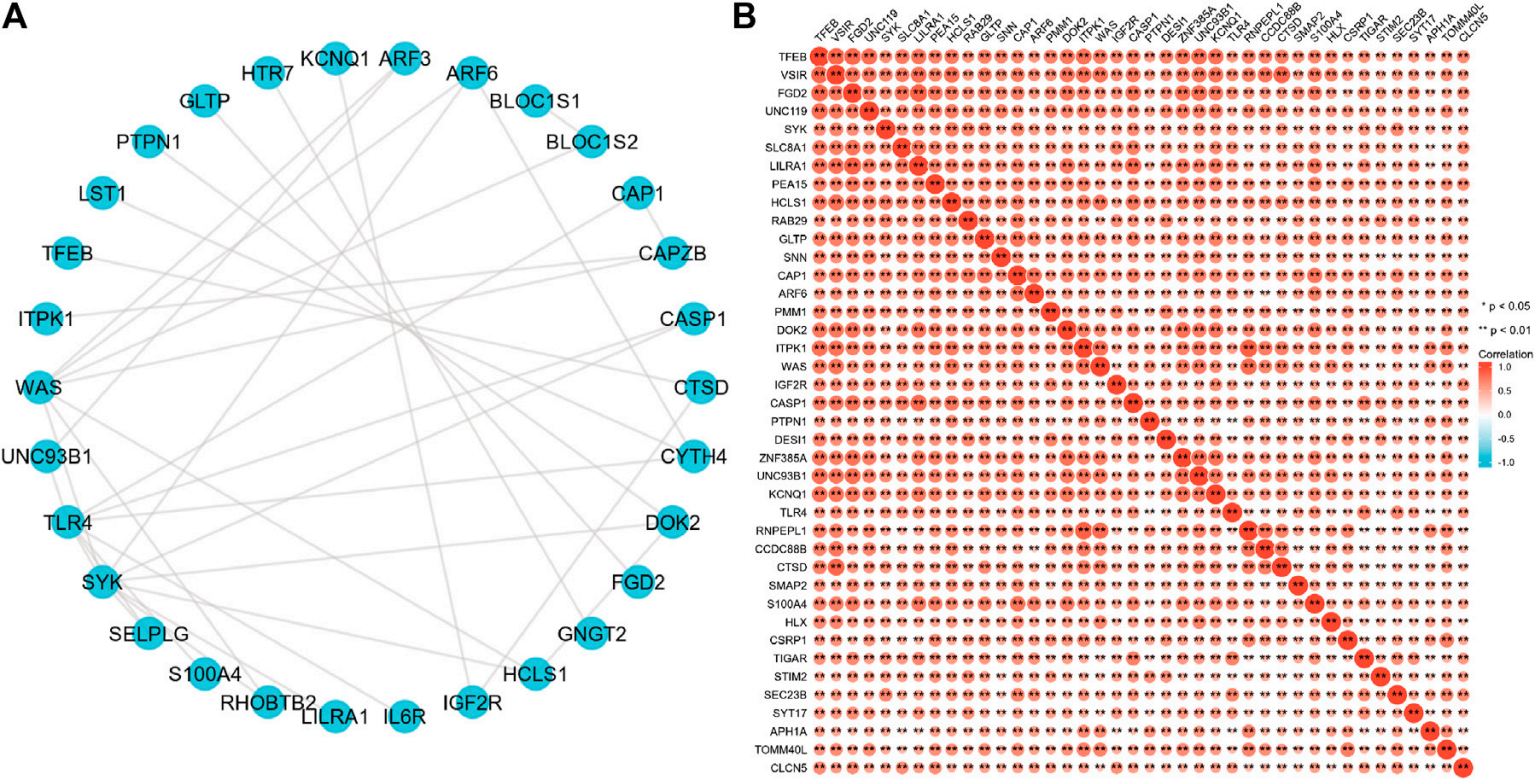
B4GALT1-associated gene interaction network **(A)** and gene co-expression matrix **(B)**.

To further explore the interactions between these 63 proteins, we constructed a PPI network for DEG using the STRING database. The most important 10 central nodes are identified by the MCC algorithm using cytoHubba. We found more robust interaction networks between these proteins and that these genes were explicitly associated with negative regulation of the apoptotic signaling pathway (Figure 7A, Supplementary Figure S3). Gene co-expression analysis showed that most of the proteins in the network showed a strong positive correlation (Figure 7B).

### B4GALT1 promoted proliferation, suppressed apoptosis of LAML cells

**FIGURE 8:**
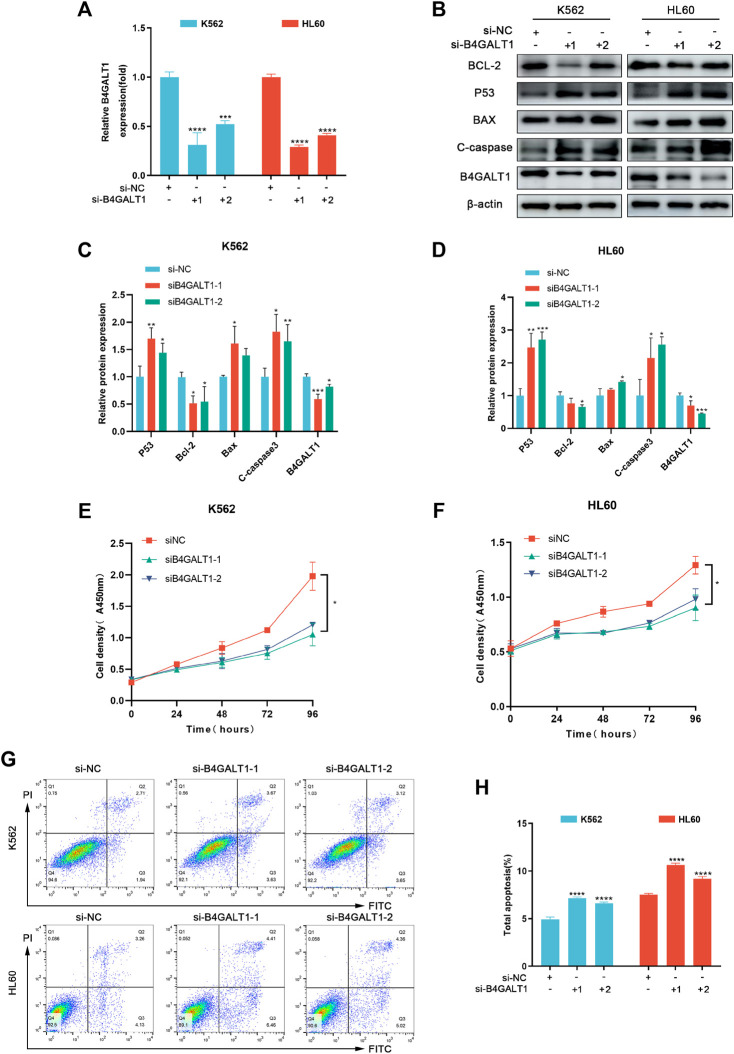
B4GALT1 suppressed apoptosis of LAML cells. **(A)** The expression of B4GALT1 was determined by qRT-PCR in B4GALT1 knockdown LAML cells. **(B–D)** The apoptosis of LAML cells was measured by western blot. **(E–F)** CCK-8 was used to detect the proliferation ability of K562 cells **(E)** and HL60 cells **(F)**. **(G–H)** The apoptosis of LAML cells was measured by staining with Annexin V/PI. NS:*p* ≥ 0.05; *, *p* < 0.05; **, *p* < 0.01; ***, *p* < 0.001.

To further investigate the regulatory role of B4GALT1 on apoptosis, we used B4GALT1-targeted siRNA sequences to interfere with the expression of B4GALT1. The results showed that the expression of B4GALT1 was significantly decreased (Figures 8A–D). Then, CCK-8 was applied to determine the effect of B4GALT1 on cell viability. The results showed that knockdown of B4GALT1 significantly inhibited the viability of K562 and HL60 cells (Figures 8E,F). Subsequently, apoptosis was determined by flow cytometry and western blot. We found that knockdown of B4GALT1 in K562 and HL60 cells promoted their apoptosis (Figures 8G,H). In conclusion, these results suggest that B4GALT1 affects the proliferation of LAML cells by regulating apoptosis.

### Correlation between B4GALT1 expression and immune infiltration

**FIGURE 9:**
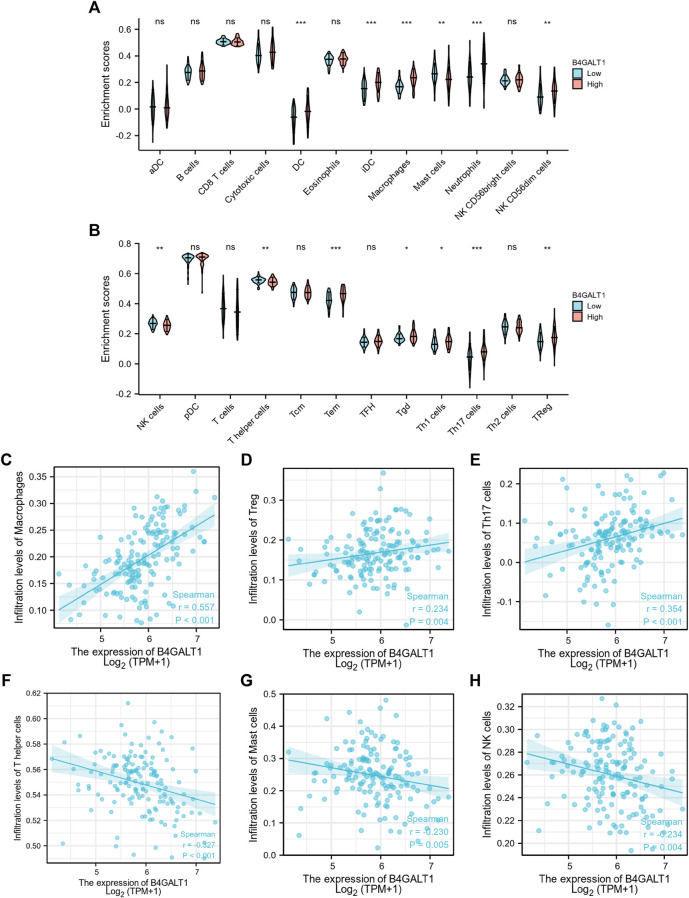
Correlation between the infiltration level of 24 immune cells and the expression level of B4GALT1. **(A-B)** Differential distribution of immune cells in patients with high B4GALT1 expression and low B4GALT1 expression. **(C-H)** Correlation between the expression level of B4GALT1 and immune infiltration in acute myeloid leukemia (LAML): **(C)** Macrophages, **(D)** regulatory T-cell, **(E)** Th17 cells, **(F)** T helper cells, **(G)** Mast cells, **(H)** NK cells. NS:*p* ≥ 0.05; *, *p* < 0.05; **, *p* < 0.01; ***, *p* < 0.001.

Based on Spearman correlation analysis, the expression level of B4GALT1 (TPM) is correlated with the level of immune cell infiltration (generated by ssGSEA). The results showed that the infiltration levels of Dendritic cells (DC), iDC, Macrophages, Neutrophils, NK CD56dim cells, Tem, Tgd, Th1 cells, Th17 cells, and Treg were significantly increased in patients with high B4GALT1 expression. In contrast, Mast cells, NK cells, and T helper cells were significantly decreased in patients with high expression of B4GALT1. (Figures 9A,B).

We further analyzed the correlation between the expression level of B4GALT1 and immune infiltration in LAML, and the results showed that the expression level of B4GALT1 was positively correlated with the infiltrating levels of Macrophages (r = 0.557, *p* < 0.001) (Figure 9C), Tregs (r = 0.234, *p* = 0.004) (Figure 9D), and Th17 cells (r = 0.354, *p* < 0.001) (Figure 9E). In contrast, the expression level of B4GALT1 was negatively correlated with the infiltrating levels of T helper cells (r = -0.327, *p* < 0.001) (Figure 9F), Mast cells (r = -0.327, *p* = 0.005) (Figure 9G), and NK cells (r = -0.234, *p* = 0.004) (Figure 9H).

## Discussion

As an essential component of cells, glycan chains are widely involved in cell adhesion, growth, differentiation, proliferation, apoptosis, and signal transduction through protein glycosylation modifications. Disorders of glycosylation can contribute to the development and progression of many tumors. It was shown that the expression and activity of Beta-1,4-galactosyltransferase one correlated with the malignancy of various tumors, including hepatocellular carcinoma, breast cancer, glioblastoma, and lung cancer (Zhu et al., 2005; Choi et al., 2012; Villegas-Comonfort et al., 2012; Tang et al., 2013; Wang et al., 2020). However, there are fewer studies on B4GALT1 in LAML. Our study used gene expression data from TCGA for bioinformatics analysis to evaluate B4GALT1’s potential mechanism and role in LAML. We have observed that the high expression of B4GALT1 in LAML is associated with poorer OS.

Meanwhile, high B4GALT1 expression was associated with a low incidence of favorable genetic abnormalities t (8; 21) and t (15; 17). Moreover, B4GALT1 has some reference value distinguishing LAML patients from healthy individuals. Therefore, B4GALT1 may become a new biomarker for LAML patients. Notably, to explain the potential molecular mechanisms by which B4GALT1 affects LAML prognosis, we performed GO and KEGG pathway analysis on 63 genes associated with B4GALT1. The results showed that B4GALT1 was significantly associated with the negative regulation of the apoptotic signaling pathway. These findings suggest that B4GALT1 is not only a new adverse prognostic factor but can also influence the pathways involved in LAML development and progression and may be a promising therapeutic target.

Chemotherapy is currently the preferred clinical treatment option for LAML. The widespread use of standard therapies can achieve complete remission (CR) in approximately 60% of patients at initial treatment. However, approximately 76% of patients eventually relapse or die, suggesting that treatment resistance has become a significant barrier to LAML prognosis (Tallman et al., 2005; Dohner et al., 2010; Dohner et al., 2015; Komanduri and Levine, 2016). Studies have shown that an important cause of tumor cell resistance to conventional anticancer drugs and some targeted therapies is an evasion of apoptosis (Aleksakhina et al., 2019). Previous studies have focused on BCL-2 family members in LAML (Sillar and Enjeti, 2019). However, resistance to drugs based on the BCL-2 family as targets has emerged during clinical use, which is partly attributed to the functional compensation of non-targeted anti-apoptotic molecules (Mazumder et al., 2012; Pan et al., 2015; Pan et al., 2017; Guieze et al., 2019; Nechiporuk et al., 2019). Discovering new candidates is critical to clinical treatment. Our study found that B4GALT1 expression was elevated in drug-resistant LAML cell lines. To further analyze the regulatory mechanisms of B4GALT1 affecting LAML progression and prognosis, we identified 63 overlapping genes as our hub genes and performed GO and KEGG functional enrichment analysis.

Interestingly, the negative regulation of the apoptotic signaling pathway was significantly enriched in the B4GALT1 high expression group. We further silenced B4GALT1 and showed significant apoptosis in LAML cell lines after B4GALT1 silencing. In addition, we found that B4GALT1 was significantly elevated in patients with non-M3 leukemia and cytogenetic risk (intermediate/poor). The Cox regression analysis suggests that B4GALT1 may have the ability to be an independent predictor of poor prognosis in LAML. Thus, our data suggest that B4GALT1 may act as a non-targeted anti-apoptotic molecule to regulate LAML progression and drug resistance by negatively regulating apoptosis, but further experimental validation is needed to demonstrate the effect of B4GALT1 on LAML development.

More and more essential and clinical studies have shown that the tumor microenvironment (TME) plays a crucial role in tumors’ occurrence, development, and metastasis. The latest study report shows that B4GALT1 is a newly discovered PD-L1 glycosyltransferase, and in triple-negative breast cancer, RBMS1 can bind to the 3′-UTR of B4GALT1 to stabilize its mRNA. Subsequently, highly expressed B4GALT1 can promote PD-L1 glycosylation and increase PD-L1 protein stability, weakening the body’s antitumor immune response and promoting tumor immune escape (Zhang et al., 2022). However, in LAML, the relationship between B4GALT1 and immune infiltration has not been reported. Therefore, we investigated the correlation between B4GALT1 and immune infiltration in LAML. We found that the abundance of Macrophages, Treg cells, and Th17 cells increased significantly in the high expression group of B4GALT1.

In contrast, the abundance of T helper cells, Mast cells, and NK cells decreased significantly. Tumor-associated macrophages (TAMs), one of the most critical subpopulations of the many tumor-infiltrating immune cells, play a crucial role in the interaction between the immune system and tumor cells. In most human tumors, tumor-associated macrophage infiltration and upregulation of their associated gene expression severely affect tumor prognosis and treatment outcome (Hu et al., 2016). Studies have reported that TAMs promote tumor angiogenesis and hematogenous cell metastasis by secreting many pro-angiogenic factors, such as vascular endothelial growth factor VEGF (Lin et al., 2006; DeNardo et al., 2009). TAM can also produce immunosuppressive factors such as IL-10, TGFβ, and PGE2, which can promote tumorigenesis and development by suppressing the antitumor immune response, among which IL-10 can significantly reduce the effect of antitumor therapy by suppressing the antitumor immune response of chemotherapy drugs (Ruffell et al., 2014). Large amounts of TAMs secrete pleiotrophin (PTN) protein in glioblastoma tumors. Glioblastoma tumor stem cells (CSCs) have large amounts of its receptor, PTPRZ1, which activate a series of signaling pathways that produce CSCs and maintain their malignant behavior, promoting tumor growth and progression and leading to increased mortality in patients (Shi et al., 2017).

Moreover, Treg cells and Th17 cells also play an essential role in the immune escape of tumors. Treg cells are one of the most critical immunosuppressive components of the bone marrow environment (Ustun et al., 2011). Studies have shown that the proportion of Tregs in peripheral blood (PB) and bone marrow (BM) in patients with LAML increases, leading to a poor prognosis for patients (Szczepanski et al., 2009; Yang and Xu, 2013). Recent studies have shown that Tregs, an essential component of the acute myeloid leukemia (LAML) microenvironment, secretes IL-10 that interacts with IL-10 R on the surface of cancer cells and further activates the PI3K/AKT signaling pathway, promoting stemness of leukemic stem cells and the development of leukemia (B4GALT1u et al., 2021). In addition, it has been reported that in LAML, Th17 cells secrete TNF-alpha and activate CD4^+^CD25^+^ Treg cells through TNFR2 receptors, leading to increased expression of tumor necrosis factor-alpha and Treg cells, which in turn leads to an immunosuppressed state in patients (Wang et al., 2018).

Furthermore, natural killer cells (NK) are the first line of defense of the body’s immune system. Unlike T and B-cell, they can rapidly kill tumor- or virus-infected cells without antigenic stimulation (Mandal and Viswanathan, 2015). Therefore, based on our findings and the above-reported studies, how B4GALT1 mediates immune escape in LAML remains further investigated.

Although our study can bring new insights into the connection between B4GALT1 and LAML, there are some limitations. First, we only analyzed the data in the database and did not collect a large sample ourselves to validate our conclusions, which may lead to biased results. Therefore, to improve the reliability of our results, we need to collect a wider sample of data to validate our conclusions. Second, the amount of our experimental data was too small, resulting in insufficient validation of our conclusions. We need further experiments to validate the biological functions of B4GALT1 *in vitro* and *in vivo*. In addition, although our data suggest that B4GALT1 may be involved in antitumor immunosuppression in LAML, further experiments are needed to verify the relationship between the two. Based on the limitations of the above studies, our laboratory has started to develop several programs.

## Conclusion

Our study found a significant upregulation of B4GALT1 expression in LAML, which was also associated with a poor prognosis. High expression of B4GALT1 may mediate chemotherapy resistance in LAML. In addition, B4GALT1 may influence LAML progression by regulating apoptosis and immune infiltration. Therefore, B4GALT1 is expected to be a potential biomarker for LAML diagnosis and prognosis.

## Data Availability

The datasets presented in this study can be found in online repositories. The names of the repository/repositories and accession number(s) can be found in the article/Supplementary Material.

## Abbreviations

LAML, acute myeloid leukemia; B4GALT1, Beta-1,4-galactosyltransferase one; TCGA, the cancer genome atlas; GTEx, Genotype-Tissue Expression; DEGs, Differentially Expressed Genes; ssGSEA, single-sample Gene Set Enrichment Analysis; GO, Gene Ontology; CC, cellular component; MF, molecular function; BP, biological process; KEGG, Kyoto Encyclopedia of Genes and Genomes; STRING, Search Tool for the Retrieval of Interacting Genes; PPI, Protein-protein interaction; ROC, receiver operating characteristic; AUC, area under the curve.
